# Optimizing *de novo* common wheat transcriptome assembly using short-read RNA-Seq data

**DOI:** 10.1186/1471-2164-13-392

**Published:** 2012-08-14

**Authors:** Jialei Duan, Chuan Xia, Guangyao Zhao, Jizeng Jia, Xiuying Kong

**Affiliations:** 1 Key Laboratory of Crop Gene Resources and Germplasm Enhancement, Ministry of Agriculture, National Key Facility for Crop Gene Resources and Genetic Improvement, Institute of Crop Science, Chinese Academy of Agricultural Sciences, No. 12 South Street, Zhongguancun, Beijing 100081, People's Republic of China; 2 College of Biology Sciences, China Agricultural University, No. 2 Yuanmingyuan West Road, Haidian District, Beijing, 100094, People's Republic of China

## Abstract

**Background:**

Rapid advances in next-generation sequencing methods have provided new opportunities for transcriptome sequencing (RNA-Seq). The unprecedented sequencing depth provided by RNA-Seq makes it a powerful and cost-efficient method for transcriptome study, and it has been widely used in model organisms and non-model organisms to identify and quantify RNA. For non-model organisms lacking well-defined genomes, *de novo* assembly is typically required for downstream RNA-Seq analyses, including SNP discovery and identification of genes differentially expressed by phenotypes. Although RNA-Seq has been successfully used to sequence many non-model organisms, the results of *de novo* assembly from short reads can still be improved by using recent bioinformatic developments.

**Results:**

In this study, we used 212.6 million pair-end reads, which accounted for 16.2 Gb, to assemble the hexaploid wheat transcriptome. Two state-of-the-art assemblers, Trinity and Trans-ABySS, which use the single and multiple k-mer methods, respectively, were used, and the whole *de novo* assembly process was divided into the following four steps: pre-assembly, merging different samples, removal of redundancy and scaffolding. We documented every detail of these steps and how these steps influenced assembly performance to gain insight into transcriptome assembly from short reads. After optimization, the assembled transcripts were comparable to Sanger-derived ESTs in terms of both continuity and accuracy. We also provided considerable new wheat transcript data to the community.

**Conclusions:**

It is feasible to assemble the hexaploid wheat transcriptome from short reads. Special attention should be paid to dealing with multiple samples to balance the spectrum of expression levels and redundancy. To obtain an accurate overview of RNA profiling, removal of redundancy may be crucial in *de novo* assembly.

## Background

The advent of next-generation sequencing technology in recent years has provided new opportunities to the field of RNA sequencing (RNA-Seq), which has emerged as a powerful tool for transcriptome study. With advances in sequencing technology, RNA-Seq, which is cost-efficient and yields sufficient information, has been widely used in both well-studied model organisms and non-model organisms in various studies, including transcript profiling, SNP discovery, and the identification of genes that are differentially expressed between samples [1-5].

For organisms with known reference genomes, mapping-first approaches have often been used for RNA-Seq analysis. Reads were first mapped to the annotated references. Then, the assembly of transcripts and the quantification of transcript expression levels were based on the mapping information. Alternatively, for those organisms lacking well-defined genomic references, these studies were typically performed using references to related species [6,7], assembled ESTs from target species [5,8] or *de novo* assembly of RNA-Seq data [9]. To use sequences from related species as references, there must be a well-studied and closely related species. Moreover, mapping reads to a related organism may result in a loss of information regarding species-specific genes, and, additionally, no overview of the target transcriptome can be generated. Assembling ESTs from the organism of interest to serve as a reference requires the existence of comprehensive EST information, and researchers may lose tissue-specific information. This loss poses a problem because the reliability of the results partly depends on the quality of EST assembly. To gain an accurate overview of the transcriptome, *de novo* assembly is crucial for downstream RNA-Seq analyses. However, *de novo* assembly of the transcriptome has some unique challenges, particularly in the case of plants, which possess a large amount of paralogs and isoforms. Assembling non-normalized transcriptomes is different from assembling normalized transcriptomes and genomes because the read depth of transcripts is uneven, which, in turn, reflects differences in expression levels. Many *de novo* assembly projects for non-model organisms have used Roche 454 pyrosequencing technology (currently about 500 bp) because the length of reads generated are much longer than the short reads (< 150 bp currently) generated by Illumina SOLEXA and ABI SOLiD technologies. However, short-read technologies are much more economical. Recently, Oases, Trinity, and Trans-ABySS have been developed specifically for RNA-Seq assembly using short sequence reads and have been applied successfully in many experiments [10-12].

Common wheat (*Triticum aestivum* L., 2*n* = 6*x* = 42) is one of the most important food crops in the world. The large genome (16 Gb), high proportion of repetitive sequences (> 80%), and hexaploid nature make complete sequencing of the wheat genome a daunting task for even state-of-the-art technologies. In addition to the identification of differentially expressed genes among different phenotypes to understand biological processes, due to the lack of high quality genomic sequence, RNA profiling is a practical approach to surveying the wheat genome. However, few wheat studies had taken advantage of RNA-Seq. Cantu *et al.*[13] assembled 1.4 million 454 reads into 30,497 contigs to study grain protein content gene in wheat. Li *et al.*[8] performed an mRNA tag analysis of wheat seedlings, rather than using assembly and mapped sequence reads to assemble public ESTs, to gain an overview of how wheat respond to H_2_O_2_ treatments. Pont *et al.*[7] assembled 934,928 reads generated from 454 sequences of normalized cDNA libraries into 37,695 sequence clusters, accounting for 20.1 Mb, to understand the polyploidization events of common wheat. Pellny *et al.*[6] utilized rice sequences and 1.5 million public ESTs as references to study the transcriptome of the developing starchy endosperm of common wheat. Trick *et al.*[5] mapped short reads from two samples to 40,349 unigene sequences (31.7 Mb) to identify SNPs. However, none of these groups performed *de novo* short read assembly from non-normalized wheat cDNA libraries.

Although RNA-Seq has been successfully used in many non-model organisms to understand transcriptome architecture and various biological processes, the results of *de novo* assembly from short reads still can be improved by using recent advances in bioinformatics, such as the method of merging single k-mer assemblies [10,12,14]. Strategies for dealing with multiple samples are an important aspect of *de novo* assembly that still lacks thorough discussion. In this study, we optimized *de novo* assembly of the hexaploid wheat transcriptome using 16.2 Gb short reads, and we documented every detail of the assembly process and discussed the effects of each assembly step to gain insight into the transcriptome assembly of non-model organisms. This study also provided comprehensive new transcript data to the wheat community.

## Results

### Illumina sequencing

To obtain a general overview of the common wheat transcriptome and an initial comparison of fertile and sterile wheat transcripts, four libraries (AF, AS, SF, SS) were constructed for paired end (PE) sequencing. AF, AS, SF, and SS were mix samples of fertile anthers, sterile anthers, fertile young spikes, and sterile young spikes of common wheat, respectively. A total number of 220.5 million 90 bp PE reads accounting for 20.1 Gb of raw data were generated for the four libraries. After trimming the low-quality part at the 3’ end of each read and filtering out singleton reads and reads less than 25 bp in length, 212.6 million PE reads (16.2 Gb) were used for downstream analyses (Table 1). The GC content of raw reads dropped from about 51% to about 40%, suggesting that our data may not have had good coverage across parts of the wheat transcriptome with high GC content (Table 1).

**Table 1:** Statistics of trimmed reads

**Libraries**	**Total reads**	**Total nucleotides (nt)**	**Average length**	**GC percentage**
AF	33,067,246 (1.5%)	2,426,965,166 (23.3%)	73.4	39.15% (52.7%)
AS	80,879,822 (4.6%)	6,204,420,998 (18.7%)	76.7	40.03% (50.8%)
SF	32,678,050 (1.3%)	2,410,831,266 (22.8%)	73.8	38.95% (51.9%)
SS	65,967,360 (4.5%)	5,148,749,102 (17.1%)	78.0	40.48% (50.3%)

The numbers in the parentheses indicated the trimmed proportions of reads, bases and the GC content before trimming.

### Different strategies of *de novo* assembly

The greatest concern for plant transcriptome *de novo* assembly is the misassembly of a large amount of paralogs and diverse alleles in plants. Considering the fact that common wheat has three sub-genomes, to gain the optimal assembly, several assembly strategies were used and their performance in assembling the wheat transcriptome was compared. We chose two state-of-the-art de Bruijn graph assemblers, Trinity [11] and Trans-ABySS [12], which use single k-mer and multiple k-mer methods to generate assemblies, respectively. Trinity is reported to be efficient in recovering full-length transcripts and spliced isoforms [11], whereas Trans-ABySS is reported to yield optimal overall assembly covering wide transcript expression levels by merging multiple individual k-mer assemblies [12]. Moreover, because we had four libraries reflecting different phenotypes and tissues, we evaluated the performance of assembling each library separately and then merged them or assembled all libraries at the initial stage. For each of these two programs, we performed seven assemblies that can be divided into the following three categories: assembly of all four samples as a whole (designated as AFSFASSS), assembly of samples with the same phenotype (designated as AFSF and ASSS) and assembly of each of the four samples individually (designated as AF, SF, AS, and SS). AFSF and ASSS were then merged to form the final assembly (designated as AFSF + ASSS), as well as the four individual assemblies of AF, SF, AS, and SS (designated as AF + SF + AS + SS).

### Statistics of pre-assemblies

**Figure 1:**
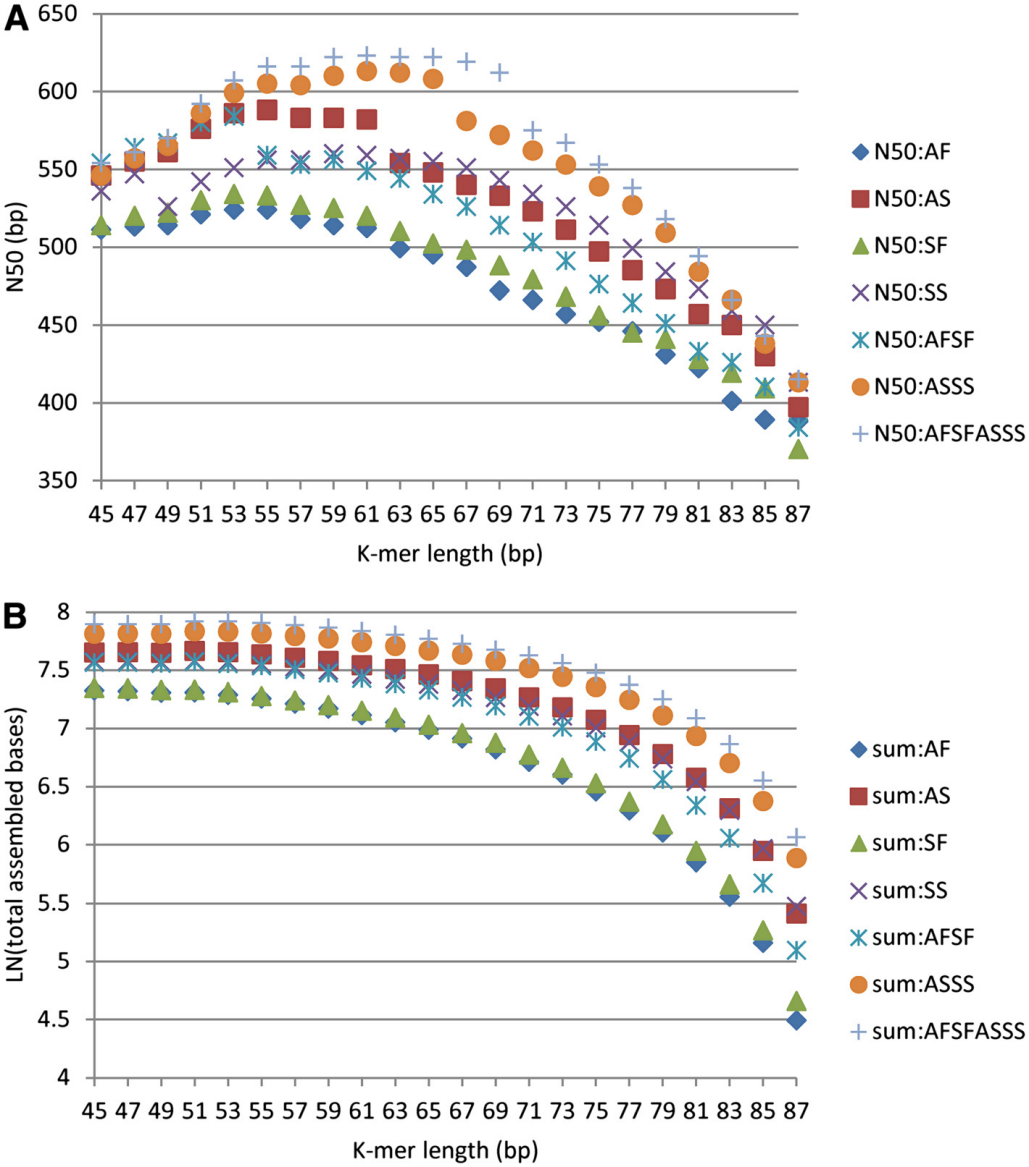
**N50 and total length of the assemblies produced by ABySS with different k-mers.** Every odd number starting from 45 to 87 were used as k-mer sizes, and different assembly strategies were compared. For example, AF refers to the use of reads from the AF sample for assembly, whereas AFSFASSS refers to the use of reads from all four samples together. **A**) N50, **B**) total length. The total length of each assembly is shown on a logarithmic scale.

For each of the Trans-ABySS assemblies, we used k-mer lengths from every odd number starting from 45 to 87. To facilitate the subsequent merging step, the scaffolding option, which introduces Ns into the assembly results, was turned off. For every k-mer length, the more reads that were used, the larger were the N50 and the total sum of assembly (Figure 1A, 1B). The largest N50 of individual k-mer assemblies for AFSFASSS, AFSF, ASSS, AF, AS, SF, and SS was 623 bp (k-mer length of 61), 584 bp (53), 613 bp (63), 524 bp (53), 588 bp (55), 534 bp (53), and 560 bp (59), respectively. After merging individual k-mer assemblies, the N50 slightly increased for all seven assemblies (Table 2). In contrast to the Trans-ABySS results, Trinity assemblies had much larger N50 and more contigs that were longer than 1 kb. Alternatively, Trans-ABySS produced much larger transcriptomes, which were on average 1.7 times larger than the results when using Trinity (Table 2).

**Table 2:** Statistics of initial assembly

	**AFSFASSS**	**AFSF**	**ASSS**	**AF**	**AS**	**SF**	**SS**
**Trinity**							
No. of contigs	227,879	110,162	198,382	69,721	133,556	76,474	126,848
Total Bases	217,485,766	89,132,635	184,623,552	3,9925,904	115,757,220	55,932,501	100,283,444
No. of contigs (> = 1 kbp)	69,402	28,016	58,759	6,817	36,825	16,409	30,694
Total Bases (in contigs > = 1 kbp)	135,112,080	47,002,017	112,433,788	9,818,210	65,495,871	25,146,108	50,760,639
Max contig length	12,488	7,710	10,506	5,723	11,199	5,670	5,294
Mean contig length	954	809	930	572	866	731	790
N50	1,370	1,060	1,323	600	1,165	903	1,015
No. of contigs in N50	46,815	25,597	41,204	20,241	29,723	19,351	30,035
**ABySS**							
No. of contigs	571,835	258,087	478,056	157,039	334,220	158,856	279,549
Total Bases	35,941,1943	152,450,028	296,430,486	88,785,112	202,564,854	90,257,267	165,879,838
No. of contigs (> = 1 kbp)	66,393	22,844	52,783	11276	33,779	11,757	25,615
Total Bases (in contigs > = 1 kbp)	88,342,878	29,539,332	69,895,282	14,500,455	44,327,763	14,876,118	33,225,449
Max contig length	8,216	4,077	5,694	5,031	6,687	4,725	4,804
Mean contig length	628	590	620	565	606	568	593
N50	686	634	674	597	653	604	637
No. of contigs in N50	178,083	82,822	149,467	51,187	105,491	51,861	89,188

Contigs less than 300 bp were excluded.

### Merging of individual sample assemblies, redundancy reduction and scaffolding

Assemblies from individual samples were merged using cd-hit-est with 100% identity [15] to form the overall assemblies. Contigs representing non-sample-specific genes existing across multiple samples were merged into single contigs. After merging individual samples, both the number and the total summed length of contigs had been reduced. The most redundant assembly was Trans-ABySS_AF + SF + AS + SS, because the number of contigs had been reduced by 22.54%. Assemblies produced by Trans-ABySS contained many more non-sample-specific contigs. The merged proportion of the four-sample-merging approach was higher than that of merging two assemblies (AFSF, ASSS, Table 3, Additional file 1: Figure S1, detailed statistics can be found in Additional file 2: Table S1). After merging separate sample assemblies, we had the following six pre-assemblies: three assemblies from Trinity and three from Trans-ABySS.

**Table 3:** Statistics of each assembly step

	**AFSFASSS**		**AFSF + ASSS**		**AF + SF + AS + SS**	
	**Trinity**	**Trans-ABySS**	**Trinity**	**Trans-ABySS**	**Trinity**	**Trans-ABySS**
**After merging**^a^						
No. of contigs	227,879	571,835	297,319	636,921	379,837	720,131
Total Bases	217,485,766	359,411,943	267,708,300	398,478,666	298,801,419	444,024,673
N50	1,370	686	1,243	683	1,000	670
No. of contigs in N50	46,815	178,083	64,054	199,771	89,338	228,145
**After deduplication**						
No. of contigs	165,174	152,963	179,876	138,487	191,858	128,990
Total Bases	139,479,895	117,699,005	151,369,559	104,950,724	151,376,565	94,829,611
N50	1,146	935	1,134	914	1,016	878
No. of contigs in N50	34,500	39,253	38,937	35,969	44,201	33,877
**After scaffolding**						
No. of contigs	162,090	152,694	176,983	138,452	188,653	129,464
Total Bases	139,459,722	120,117,056	151,354,485	107,614,336	151,363,021	97,866,545
N50	1,177	960	1,161	942	1,041	907
No. of contigs in N50	33,941	39,124	38,409	35,915	43,481	33,927
Proportion of Ns	0.12%	0.13%	0.12%	0.13%	0.12%	0.13%
GC content	47.93%	48.69%	48.18%	48.76%	48.51%	48.81%

^a^ Trinity_AFSFASSS and Trans-ABySS_AFSFASSS had not been merged.

Detail statistics can be found in Additional file 2. Contigs less than 300 bp were excluded.

**Figure 2:**
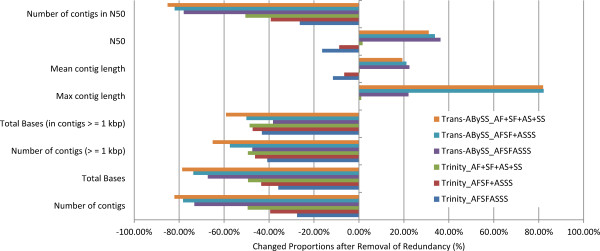
**Effects of removal of redundancy.** The influence of removal of redundancy on the six assemblies, shown as a changed proportion.

Contigs that overlapped with a minimum length of 50 bp and minimum identity of 99% were collapsed into single contigs using GICL to further remove redundancy [16]. After this step, a large proportion of contigs had been merged (Figure 2, Table 3). Before deduplication (clustering related contigs to remove redundancy), assemblies produced by Trans-ABySS had a much greater number of contigs and total summed length than those of the counterpart Trinity assemblies. After the removal of redundancy, both the number of contigs and the summed lengths were lower than those of the counterpart Trinity assemblies (Table 3). Moreover, this deduplication step significantly increased the N50 of the three Trans-ABySS assemblies (*p* = 0.003). Merging individual assemblies, including multiple samples or multiple k-mer assemblies, introduced more redundancy.

**Figure 3:**
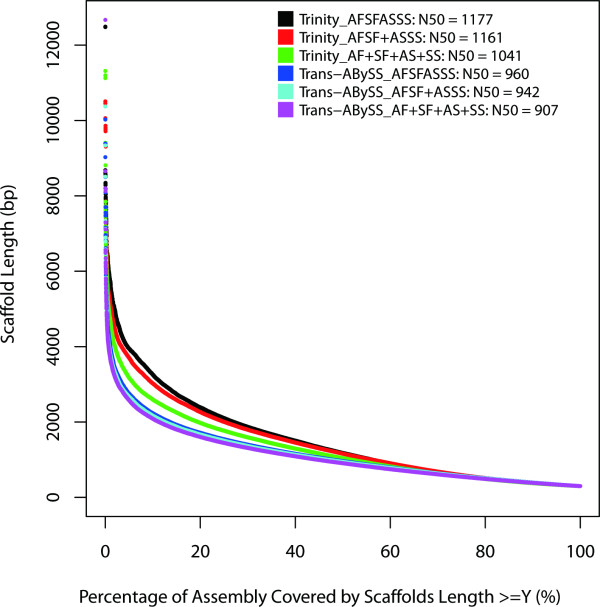
**Cumulative scaffold lengths generated by different assembly programs and strategies.** For the six assemblies, scaffolds shorter than 300 bp were filtered.

To reduce fragmental degree, contigs were joined using read pair information as implemented by SSPACE [17]. After scaffolding, the N50 of all six assemblies had been slightly increased by approximately 2.5% (Additional file 1: Figure S1, Table 3, Figure 3).

### Assessment of novelty and redundancy

**Figure 4:**
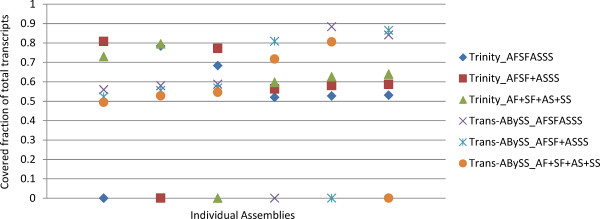
**Pairwise comparisons between the six assemblies.** Assemblies were compared in a pair-wise fashion using BLAT, and the proportions covered are shown.

To determine whether the different assembly sizes and contig numbers were mainly due to novel sequences in each assembly or redundancy, pairwise comparisons of the six assemblies were performed using BLAT [18]. The novelty and redundancy of each assembly were determined by the proportion of base coverage by the other five assemblies with a high threshold (a minimum overlap length of 50 bp and minimum identity of 99%). Pairwise comparisons showed that the Trinity_AF + SF + AS + SS assembly had the most novel bases, whereas the Trans-ABySS_AFSF + ASSS assembly had the fewest. Trinity assemblies had more novel sequences (on average, 79.15% of bases were covered) than those of Trans-ABySS (85.21%, Figure 4, Additional file 3: Table S2). Assemblies produced by the same assembler were covered more frequently by each other (Figure 4, Additional file 3: Table S2).

To evaluate assembly redundancy, reads from all four samples were mapped to the six assemblies. The proportion of reads mapped to more than three locations was used as an indicator of redundancy. On average, after removing redundancy and scaffolding, Trans-ABySS assemblies were less redundant than Trinity assemblies (Table 4). Similarly, the merging samples approach resulted in more redundancy, whether using Trinity or Trans-ABySS. The most redundant assembly was Trinity_AF + SF + AS + SS, of which 10.56% of its mapped reads had more than three hits. The contig number and total size of Trinity_AF + SF + AS + SS were also the largest among the six assemblies.

**Table 4:** Statistics of reads mapped to assemblies

**Proportion of reads**	**Trinity**			**Trans-ABySS**		
	**AFSFASSS**	**AFSF + ASSS**	**AF + SF + AS + SS**	**AFSFASSS**	**AFSF + ASSS**	**AF + SF + AS + SS**
AF aligned	83.83%	87.53%	89.17%	93.62%	92.44%	91.48%
aligned more than 3 hits	4.18%	8.12%	9.63%	2.93%	3.89%	4.91%
uniquely aligned	51.85%	48.67%	48.48%	71.74%	67.46%	63.89%
AS aligned	82.21%	86.50%	88.27%	92.82%	91.85%	90.73%
aligned more than 3 hits	4.23%	8.35%	10.45%	3.00%	3.85%	4.93%
uniquely aligned	50.67%	48.48%	47.70%	70.94%	66.89%	62.74%
SF aligned	80.06%	86.13%	88.61%	93.55%	92.23%	91.41%
aligned more than 3 hits	3.88%	8.30%	11.46%	3.92%	4.79%	6.08%
uniquely aligned	50.48%	48.73%	47.06%	71.63%	67.09%	62.58%
SS aligned	74.98%	86.39%	88.47%	92.67%	91.68%	90.81%
aligned more than 3 hits	3.81%	7.83%	10.71%	3.19%	3.98%	5.07%
uniquely aligned	46.73%	51.45%	49.93%	72.35%	68.42%	64.23%
All aligned	79.89%	86.57%	88.52%	93.01%	91.95%	90.98%
aligned more than 3 hits	4.04%	8.15%	10.56%	3.19%	4.04%	5.15%
uniquely aligned	49.60%	49.47%	48.41%	71.61%	67.48%	63.35%

### Assessment of sample specificity

To evaluate the performance of assembling all libraries at the initial stage and assembling each library separately and then merging them for each of the two assemblers, we performed three assemblies, AFSFASSS, AFSF + ASSS, AF + SF + AS + SS, representing the assembly of all samples, samples with the same phenotype, and individual samples at the initial stage, respectively. The purpose of this evaluation was to determine whether AFSFASSS and AFSF + ASSS lost a considerable amount of sample-specific transcripts or if AFSF + ASSS and AF + SF + AS + SS contained more redundant transcripts and more fragmental transcripts.

Reads from four samples were mapped to the six assemblies separately. With the exception of Trinity_AFSFASSS, the proportions of reads for the four samples mapped to the other five assemblies were equivalent, indicating that the assemblies did not favor certain samples. As for Trinity_AFSFASSS, the proportion of mapped reads from the SS sample was only 74.98%, and those for AF, AS, and SF were all above 80%, suggesting that Trinity_AFSFASSS was less represented in the SS sample. In the instance of the AF sample, the proportion of reads mapped to Trans-ABySS_AFSFASSS, Trans-ABySS_AFSF + ASSS, and Trans-ABySS_AF + SF + AS + SS was 93.62%, 92.44%, and 91.48%, respectively, suggesting that for Trans-ABySS assemblies, the more assemblies that were merged, the less reads were mapped, which differs from Trinity assemblies. Merging individual Trinity assemblies increased mappable reads.

### Assessment of continuity and accuracy

A full-length common wheat cDNA dataset from TriFLDB was used to evaluate assembly continuity [19]. This dataset contained 6,137 sequences ranging from 131 bp to 8,930 bp in length (N50 of 1,966 bp). Six assemblies were used to BLAT against these full-length transcripts. The proportion of numbers and bases of full-length transcripts covered by each of the six assemblies was calculated and are shown in Table 5. Approximately 90% of full-length cDNAs were matched by the six assemblies separately, and nearly all matched assembled transcripts covering over 90% of the full-length cDNA in length. Trinity outperformed Trans-ABySS in assembly continuity (Table 5), and for both assemblers, merging individual assemblies decreased contig length, particularly for Trans-ABySS.

**Table 5:** Comparisons between full-length cDNA transcripts and assembled transcripts

**6,137 fl-cDNA transcripts**	**Trinity**			**Trans-ABySS**		
	**AFSFASSS**	**AFSF + ASSS**	**AF + SF + AS + SS**	**AFSFASSS**	**AFSF + ASSS**	**AF + SF + AS + SS**
% of fl-cDNA hit	92.94%	92.83%	92.62%	91.33%	90.39%	89.69%
(% of bases covered)	82.84%	82.64%	81.68%	79.52%	76.71%	74.09%
% of fl-cDNA hit with at least 90% of its length	92.06%	92.05%	92.13%	90.83%	89.73%	88.90%
(% of bases covered)	82.08%	82.02%	81.14%	79.24%	76.40%	73.79%

Another metric of assembly performance is transcript accuracy. To gain a general overview of transcript accuracy, a custom EST library, constructed using the recurrent parent Chinese Spring (CS) containing 51,449 Sanger-sequenced ESTs ranging from 100 bp to 922 bp in length (N50 of 634 bp) was used (Zhao GY *et al.*, unpublished). With the threshold minimum of 98% base identity, Trans-ABySS_AFSFASSS covered 81.52% CS ESTs bases, which was greater than the other five assemblies. On average, Trans-ABySS assemblies were much more similar to the CS ESTs than those of Trinity (Table 6).

**Table 6:** Comparisons between Chinese Spring cDNA transcripts and assembled transcripts

**51,449 Chinese Spring (CS) ESTs**	**Trinity**			**Trans-ABySS**		
	**AFSFASSS**	**AFSF + ASSS**	**AF + SF + AS + SS**	**AFSFASSS**	**AFSF + ASSS**	**AF + SF + AS + SS**
% of CS ESTs hit	92.64%	93.97%	93.60%	92.89%	91.65%	90.63%
(% of bases covered)	85.82%	87.97%	87.91%	87.73%	85.72%	83.99%
% of CS ESTs hit with at least 98% of identity	51.56%	65.21%	76.07%	73.98%	71.58%	69.19%
(% of bases covered)	59.46%	65.15%	69.54%	81.52%	79.25%	77.08%

### Comparisons with public wheat ESTs

To compare the six assemblies to other publicly available transcript data, all common wheat ESTs deposited at dbEST/GenBank were downloaded (1,073,845 ESTs with an N50 of 639 bp, 2012.01.24). Similarity searches against this EST dataset were performed (Table 7). The proportions of public common wheat ESTs bases covered by these six assemblies were all approximately 80%, and the highest one was Trans-ABySS_AFSFASSS. In general, approximately 80% of EST bases were covered by these six assemblies. However, the proportions of the six assemblies covered by this EST dataset varied, with the lowest being 57.09% from Trinity_AFSFASSS to the highest being 75.90% from Trans-ABySS_AF + SF + AS + SS.

**Table 7:** Comparisons between public ESTs and assembled transcripts

	**Trinity**			**Trans-ABySS**		
	**AFSFASSS**	**AFSF + ASSS**	**AF + SF + AS + SS**	**AFSFASSS**	**AFSF + ASSS**	**AF + SF + AS + SS**
% of ESTs hit^a^	87.83%	88.89%	88.86%	88.64%	87.91%	87.29%
(% of bases covered)	77.76%	79.76%	80.04%	80.49%	79.50%	78.61%
% of assembled transcripts hit^b^	59.54%	65.43%	69.82%	78.05%	80.58%	80.78%
(% of bases covered)	57.09%	62.59%	66.09%	70.91%	74.24%	75.90%

^a^ Proportion of public ESTs matched by assembled transcripts.

^b^ Proportion of assembled transcripts matched by public ESTs.

### Size of wheat transcriptome

The sizes of the six assemblies varied from 97.9 Mb to 151.4 Mb. These transcripts were mapped to a draft genomic sequence of *Ae*. *tauschii*, which is the diploid progenitor of common wheat (Jia *et al.*, unpublished). We selected only 237,145 scaffolds ranging from 300 bp to 720,471 bp in length from the D genome sequence to perform the alignments. Assembled transcripts from the six assemblies were aligned to the draft reference sequence using BWASW with default parameters (a mode of BWA) [20], and the numbers of covered bases for the diploid sequence were calculated as shown in Table 8. On average, approximately two transcripts aligned at a single locus for all six assemblies, whereas the coverage range differed dramatically. Trinity_AFSFASSS covered about 62.1 Mb for this diploid draft genome, which was the most among the six assemblies (Table 8).

**Table 8:** **Statistic of transcripts aligned to the draft diploid *Ae*. *tauschii *genome**

	**Trinity**			**Trans-ABySS**			**Public ESTs**	**Trinity_AFSFASSS_2**^**nd**^
	**AFSFASSS**	**AFSF + ASSS**	**AF + SF + AS + SS**	**AFSFASSS**	**AFSF + ASSS**	**AF + SF + AS + SS**		
Mean coverage	1.9	2.2	2.3	2	2.1	2.1	11.0	2.9
Coverage range	1 - 279	1 - 339	1 - 254	1 - 26	1 - 24	1 - 24	1 - 10,122	1 - 658
Bases covered	62,103,048	58,923,609	56,105,411	51,499,767	45,678,989	41,571,199	46,506,263	63,042,809

Assembled transcripts were aligned to the draft genomic sequence. For the covered regions of the reference by assembled transcripts, mean coverage indicated how many transcripts covered on average for a certain region.

## Discussion and conclusions

In this study, we constructed six wheat transcriptome assemblies using different assemblers and merging strategies on 16.2 Gb PE data. We divided the assembly processes into the following four steps: pre-assembly, merging samples, removal of redundancy, and scaffolding. We recorded the effects of each step in detail. The yielded six assemblies had promising N50 (Table 3), continuity (Table 5), and accuracy (Table 6).

### Performance comparisons between Trinity and Trans-ABySS

Trinity, which uses the single k-mer method, outperformed Trans-ABySS in assembly continuity. The average N50 of the three Trinity assemblies was 16.7% longer than that of Trans-ABySS (Table 3). All three Trinity assemblies covered more bases of full-length cDNA than their counterpart Trans-ABySS assemblies (Table 5). After the same procedures, Trans-ABySS assemblies, which were produced by the multiple k-mer method, were less redundant (Table 4) and covered a greater proportion of existing Sanger-derived ESTs (Tables 6, 7), representing a good spectrum of expression levels.

Pairwise comparisons of these six assemblies suggested that assemblies produced by the same assembler were more similar to each other. There was a tradeoff between specificity and sensitivity based on the choice of k-mer size. Assemblies with larger k-mers had fewer spurious overlaps but lower coverage. Low-expression genes were assembled more effectively with small k-mer sizes, leading to the assembly of numerous and highly fragmented transcripts, whereas high-expression genes were assembled more effectively with large k-mer sizes, emphasizing contiguity [12,14]. For Trinity, we used the default k-mer size of 25, while Trans-ABySS used every odd number starting from 45 to 87. To test whether the different k-mer sets used by Trinity and Trans-ABySS caused the low level of overlapping regions, we reassembled an assembly with all four samples together using a single k-mer size of 25, as implemented by ABySS version 1.2.5 [21], named ABySS_AFSFASSS_k25. When ABySS_AFSFASSS_k25 was compared with Trinity_AFSFASSS, 40.9% of Trinity_AFSFASSS bases were covered, which was lower than the 56.0% of bases covered by Trans-ABySS_AFSFASSS (Figure 4, Additional file 3: Table S2). This suggested that when using the same k-mer size of 25, ABySS and Trinity still had a large proportion of unique bases. For the initial assemblies, Trans-ABySS produced assemblies that had more contigs that were much larger than those of Trinity (Table 2). For this dataset (mean reads length of about 75 bp), both Trinity and Trans-ABySS had good performance, and the majority of reads were able to map to the six final assemblies, particularly for the Trans-ABySS_AFSFASSS (Table 4).

### Merging assemblies of individual samples

RNA-Seq analyses often deal with multiple samples. In this study, we compared the performances of different assemblers and merging strategies. The greatest concern for assembling all samples at the initial step is the loss of sample-specific transcripts. With the exception of Trinity_AFSFASSS, the other five assemblies, including Trans-ABySS_AFSFASSS, had equal proportions of the four samples, suggesting that using reads from all samples together may not always introduce bias from over- or under-represented samples.

For the single k-mer assembler Trinity, merging assemblies of individual samples increased the number of mappable reads (Table 4) and the proportion of bases covered from full-length cDNA and public ESTs (Tables 6, 7), suggesting that the merging strategies broadened the coverage of the assemblies produced by Trinity. Alternatively, merging Trinity assemblies slightly decreased the base coverage of full-length transcripts (Table 5) and N50 (Table 3), suggesting that merging assemblies from individual samples decreased continuity. The main defect was the substantial introduction of redundancy, as indicated by the decreased number of uniquely mapped reads and increased number of repetitively mapped reads (Table 4).

For the multiple k-mer assembler Trans-ABySS, merging assemblies from individual samples had the following shortcomings: shorter N50 (Table 3), fewer number of mappable reads, reduced proportions of bases covered from full-length cDNA and public ESTs, and slightly increased redundancy (Table 4).

### Removal of redundancy

A large assembly including transcripts across a wide range of expression levels but containing many redundant contigs/scaffolds could be worse than a small assembly only contain unique bases. Redundancy can pose difficulties in the downstream analysis.

After merging assemblies from individual samples, the overall contig numbers from these six assemblies were still very high (ranging from 227,879 to 720,131), even when considering the various transcript isoforms (Table 3, Additional file 2: Table S1). The reason might be sequencing errors and potential sequence variations among individual plants. Thus, we implemented a clustering step to remove redundancy. A major concern of removing redundancy is the mis-assembly of highly similar transcripts. Although there is no straightforward way to evaluate the performance of this step due to the lack of well-defined genomic sequences, we used two datasets to assess the parameters we used: one with 51 WRKY transcription factors detected from common wheat UniGenes, and the other with 23 groups (a total 69 sequences) of orthologous genes from A, B, and D subgenomes of hexaploid wheat, representing conserved gene families and highly similar but distinct sequences, respectively (Additional file 4: Table S3). With the thresholds of a minimum length of 50 bp and minimum identity of 99%, none of these sequences were assembled together suggesting the parameters we used in removing redundancy is proper.

After removing duplications using this strict standard, for the Trinity_AFSFASSS that was assembled without merging individual assemblies, 27.52% of its contig number and 35.87% of its total length had been reduced. To compare the effect of deduplication, we reproduced an assembly (Trinity_AFSFASSS_2^nd^) that used every procedure used for assembling the Trinity_AFSFASSS without the step that removes redundancy. Trinity_AFSFASSS_2^nd^ had an N50 of 1,385 bp contained 225,280 scaffolds ranging from 300 bp to 12,488 bp in length. Trinity_AFSFASSS_2^nd^, with a total size of 217,468,671 bp, was mapped to the draft diploid *Ae*. *tauschii* sequence using the same parameters used for Trinity_AFSFASSS. Trinity_AFSFASSS_2^nd^ covered about 63.0 Mb, which was equivalent to that of Trinity_AFSFASSS, suggesting that the removing the redundancy step that we used may not greatly reduce unique transcripts and was appropriate to use (Table 8). To further compare the effects of removing redundancy, reads from the four samples were mapped to Trinity_AFSFASSS_2^nd^. Comparing to Trinity_AFSFASSS, the total number of mappable reads slightly decreased by 1.05%. Moreover, the proportion of reads mapped to more than three locations increased from 4.04% to 12.95%, and the uniquely mapped reads were reduced from 49.60% to 31.38% (Additional file 5: Table S4). It is probably difficult for this assembly to generate accurate overall downstream RNA-Seq analyses, suggesting that proper removal of redundancy may be crucial for polyploidy plant transcriptome assembly.

### Using assembled ESTs or *de novo* assemblies as references

In general, only uniquely mapped reads can be used in common downstream RNA-Seq analyses, including SNP discovery and the identification of genes that are differentially expressed between samples. This was the reason we used mapped reads, especially the number of uniquely mapped reads as a measurement of assembly performances. Considering the large amount of common wheat cDNA that already exists in the public domain, we mapped all reads to the following two most commonly used assembled EST datasets: wheat gene index (TAGI) and wheat PUT assembly (TAPUT) [22,23] (Additional file 5: Table S4). The proportions of mappable reads, repetitively mapped reads, and uniquely mapped reads were 83.36% and 81.34%, 25.00% and 15.10%, 43.77% and 51.04% of TAGI and TAPUT, respectively. When compared with the results of *de novo* assemblies, a more accurate overview of the wheat transcriptome can be obtained using *de novo* assemblies as references, particularly the Trans-ABySS_AFSFASSS (Table 4).

The GC content of the six *de novo* assemblies was about 48% (Table 3), and those of 1,073,845 common wheat ESTs, TAGI and TAPUT were 52.33%, 52.10% and 51.68%, respectively. The lower level of GC content for these assemblies may be due to filtered reads, which were already GC content-less-presented (Table 1).

### Construction of wheat transcriptome with short reads

Due to the lack of a well-annotated genomic sequence, we could not assembly the wheat transcriptome using a mapping-first strategy. To assess the performance of assembling the wheat transcriptome using short reads, we used the existing public wheat ESTs, a custom Chinese Spring cDNA library and a draft genome sequence of *Ae*. *tauschii*, the wheat D genome progenitor.

By mapping assembled transcripts back to the draft *Ae*. *tauschii* sequence, the number of bases covered by each of these assemblies was used to compare the total number of bases constructed from each assembly. Combining the length of N50 for these assemblies from both assemblers, the more reads that were used at the initial assembling step, the more unique bases that were constructed in the final assemblies. Considering that we had used the maximum of 16.2 Gb reads at the pre-assembling step, which was already more than 100 times larger than the size of the final assemblies, further improvements in assembly may need even more reads or longer reads. The use of 16.2 Gb data has already provided promising results. Nearly all assembled transcripts, which were matched by full-length cDNA using BLAT, covered 90% of full length cDNA in length (Table 5), suggesting that the length of transcripts assembled by Illumina reads was acceptable.

On average, more than one transcript was mapped to the same locations of the draft *Ae*. *tauschii* sequence for all six assemblies (Table 8), which was expected, because the reference is diploid while the transcriptome is from hexaploid wheat.

To compare the assemblies with existing wheat resources, all of the currently available wheat ESTs were aligned to the same *Ae*. *tauschii* sequence. A total of 1,073,845 common wheat ESTs deposited in GenBank covered 46.5 Mb of reference sequences, which was less than that of four of the six assemblies (Table 8). Considering the direct comparison results between these assemblies and public ESTs (Table 7), this study also provided considerable new wheat transcript resources to the research community.

## Methods

### Plant materials

Wheat tissues were collected from Taigu/CS near-isogenic lines (NILs), which were developed by backcrossing the "sterile" donor cultivar Taigu with the recurrent parent Chinese Spring (CS). Taigu carries the dominant sterile gene *ms2*. Mixed anther samples with lengths of 0.5 – 2.5 mm were collected from fertile and sterile plants and were designated as AF and AS, respectively. Mixed young spike samples with lengths of 3.0 – 4.5 cm were collected from fertile and sterile plants and were designated as SF and SS, respectively.

### RNA extraction, library construction and Illumina sequencing

Total RNA was extracted using TRIzol reagent (Life Technologies, Grand Island, NY). The quality and quantity of RNA were examined using an Agilent 2100 Bioanalyzer (Agilent Technologies, Santa Clara, CA) before the following procedures: enrichment of mRNA by Oligo(dT), fragmentation, cDNA synthesis by random hexamer primers, size selection (200 bp) and PCR amplification, which was performed by BGI-Senzhen as described previously [9]. RNA sequencing was performed on a HiSeq 2000 (Illumina, San Diego, CA).

### Raw reads filtering

Pair-end raw reads were trimmed with the BWA trimming mode at a threshold of Q13 (*P* = 0.05) as implemented by SolexaQA version 1.10 [24]. Low-quality 3’ ends of each read were filtered. Reads that were less than 25 bp in length or were singletons were discarded.

### Pre-assembly of short reads

*De novo* assembly was performed using both Trinity and Trans-ABySS. Trinity release 20110519 was employed with the ALLPATHSLG error correction, and the paired fragment length was set to 200 bp [11]. Individual k-mer assemblies were carried out by ABySS version 1.2.5 with the scaffolding option off and contig end erosion off [21]. Trans-ABySS version 1.2.0 was used to merge the individual k-mer assemblies with default parameters [12].

### Merging, deduplication and scaffolding

Assemblies of individual samples were merged with both strands information using the accurate mode of cd-hit-est version 4.5.4 with the sequence identity threshold at 100% and a word size of 8 [15]. GICL (release date 2010-07-22) was used to remove redundancy [16]. Contigs overlapped with at least 50 bp with a minimum identity of 99% were collapsed into single contigs, and the maximum length of unmatched overhangs was set to 100 bp. *Triticum aestivum* UniGene Build 60 was downloaded from NCBI. One clone-derived sequence selected from each UniGene cluster was used to perform the domain detection. WRKY HMM was downloaded from Pfam [25]. Genewisedb module from wise2 package (version 2.4.1) was used at the threshold of 1E-8 [26]. Scaffolding pre-assembled contigs was performed using SSPACE version 1.1 [17]. The command used for SSPACE was "-x 0 -m 50 -o 20 -k 5 -n 15". The Trans-ABySS_AFSFASSS assembly with the highest mappable reads among the six assemblies was deposited in GenBank/TSA under the accession Nos. JV812965-JV999999 and JW000001-JW036047. The other five assemblies are available upon request.

### Short-read alignment

Short reads were aligned to references using BWA version 0.6.1 [27] with the default parameters, expect the seeding option was turned off to ensure that mismatches were allowed to be randomly distributed along the reads. Manipulating alignment results involved the use of SAMtools version 0.1.18 [28].

### Assembled transcripts alignment

The BWASW mode in BWA was used to align assembled transcripts into the draft diploid reference with default parameters [20]. Comparisons of transcripts were performed using BLAT version 34x12 with default parameters, and the alignment results were filtered with various demands [18]. The assembled two EST datasets used in analyses were TAGI release 12.0 and wheat PUT assembly version 163b [16,23].

## Abbreviations

PE: Pair end; CS: Chinese spring; fl-cDNA: Full-length cDNA.

## Competing interests

The authors declare that they have no competing interests.

## Authors' contributions

Conceived and designed the experiments: JD CX JJ XK. Performed the experiments: CX. Performed the bioinformatics analysis: JD. Contributed reagents/materials/analysis tools: GZ. Wrote the paper: JD JJ XK. All authors read and approved the final manuscript.

## Supplementary Material

Additional file 1 **Figure S1.** Effects of merging assemblies of different samples and scaffolding (shown as changed proportions). Click here for file

Additional file 2 **Table S1.** Detailed statistics for each assembly step. Click here for file

Additional file 3 **Table S2.** Detailed pairwise comparisons between the six assemblies. Click here for file

Additional file 4 **Table S3.** Sequences from A, B and D subgenome used in the assessment of parameters used in the step of removing redundancy. Click here for file

Additional file 5 **Table S4.** Statistics of reads mapping to the assembled EST datasets (TAGI and TAPUT). Click here for file

## References

[B1] Feng C Chen M Xu CJ Bai L Yin XR Li X Allan AC Ferguson IB Chen KS Transcriptomic analysis of Chinese bayberry (*Myrica rubra*) fruit development and ripening using RNA-Seq. BMC Genomics 2012 13 19 10.1186/1471-2164-13-19 22244270 PMC3398333

[B2] Zhang G Guo G Hu X Zhang Y Li Q Li R Zhuang R Lu Z He Z Fang X Deep RNA sequencing at single base-pair resolution reveals high complexity of the rice transcriptome Genome Res 2010 20 5 646 654 10.1101/gr.100677.109 20305017 PMC2860166

[B3] Ness RW Siol M Barrett SC De novo sequence assembly and characterization of the floral transcriptome in cross- and self-fertilizing plants BMC Genomics 2011 12 298 10.1186/1471-2164-12-298 21649902 PMC3128866

[B4] Bundock PC Eliott FG Ablett G Benson AD Casu RE Aitken KS Henry RJ Targeted single nucleotide polymorphism (SNP) discovery in a highly polyploid plant species using 454 sequencing Plant Biotechnol J 2009 7 4 347 354 10.1111/j.1467-7652.2009.00401.x 19386042

[B5] Trick M Adamski NM Mugford SG Jiang CC Febrer M Uauy C Combining SNP discovery from next-generation sequencing data with bulked segregant analysis (BSA) to fine-map genes in polyploid wheat BMC Plant Biol 2012 12 14 10.1186/1471-2229-12-14 22280551 PMC3296661

[B6] Pellny TK Lovegrove A Freeman J Tosi P Love CG Knox JP Shewry PR Mitchell RA Cell walls of developing wheat starchy endosperm: comparison of composition and RNA-Seq transcriptome Plant Physiol 2012 158 2 612 627 10.1104/pp.111.189191 22123899 PMC3271754

[B7] Pont C Murat F Confolent C Balzergue S Salse J RNA-seq in grain unveils fate of neo- and paleopolyploidization events in bread wheat (*Triticum aestivum* L.). Genome Biol 2011 12 12 R119 10.1186/gb-2011-12-12-r119 22136458 PMC3334614

[B8] Li A Zhang R Pan L Tang L Zhao G Zhu M Chu J Sun X Wei B Zhang X Transcriptome analysis of H_2_O_2_-treated wheat seedlings reveals a H_2_O_2_-responsive fatty acid desaturase gene participating in powdery mildew resistance PLoS One 2011 6 12 e28810 10.1371/journal.pone.0028810 22174904 PMC3236209

[B9] Zhang J Liang S Duan J Wang J Chen S Cheng Z Zhang Q Liang X Li Y De novoassembly and characterisation of the transcriptome during seed development, and generation of genic-SSR markers in peanut (*Arachis hypogaea* L.). BMC Genomics 2012 13 1 90 10.1186/1471-2164-13-90 22409576 PMC3350410

[B10] Schulz MH Zerbino DR Vingron M Birney E Oases: robust *de novo* RNA-seq assembly across the dynamic range of expression levels Bioinformatics 2012 28 8 1086 1092 10.1093/bioinformatics/bts094 22368243 PMC3324515

[B11] Grabherr MG Haas BJ Yassour M Levin JZ Thompson DA Amit I Adiconis X Fan L Raychowdhury R Zeng Q Full-length transcriptome assembly from RNA-Seq data without a reference genome Nat Biotechnol 2011 29 7 644 652 10.1038/nbt.1883 21572440 PMC3571712

[B12] Robertson G Schein J Chiu R Corbett R Field M Jackman SD Mungall K Lee S Okada HM Qian JQ *De novo* assembly and analysis of RNA-seq data. Nat Methods 2010 7 11 909 912 10.1038/nmeth.1517 20935650

[B13] Cantu D Pearce SP Distelfeld A Christiansen MW Uauy C Akhunov E Fahima T Dubcovsky J Effect of the down-regulation of the high *Grain Protein Content (GPC)* genes on the wheat transcriptome during monocarpic senescence. BMC Genomics 2011 12 492 10.1186/1471-2164-12-492 21981858 PMC3209470

[B14] Surget-Groba Y Montoya-Burgos JI Optimization of *de novo* transcriptome assembly from next-generation sequencing data. Genome Res 2010 20 10 1432 1440 10.1101/gr.103846.109 20693479 PMC2945192

[B15] Li W Godzik A Cd-hit: a fast program for clustering and comparing large sets of protein or nucleotide sequences Bioinformatics 2006 22 13 1658 1659 10.1093/bioinformatics/btl158 16731699

[B16] Pertea G Huang X Liang F Antonescu V Sultana R Karamycheva S Lee Y White J Cheung F Parvizi B TIGR Gene Indices clustering tools (TGICL): a software system for fast clustering of large EST datasets Bioinformatics 2003 19 5 651 652 10.1093/bioinformatics/btg034 12651724

[B17] Boetzer M Henkel CV Jansen HJ Butler D Pirovano W Scaffolding pre-assembled contigs using SSPACE Bioinformatics 2011 27 4 578 579 10.1093/bioinformatics/btq683 21149342

[B18] Kent WJ BLAT–the BLAST-like alignment tool Genome Res 2002 12 4 656 664 11932250 10.1101/gr.229202 PMC187518

[B19] Mochida K Yoshida T Sakurai T Ogihara Y Shinozaki K TriFLDB: a database of clustered full-length coding sequences from Triticeae with applications to comparative grass genomics Plant Physiol 2009 150 3 1135 1146 10.1104/pp.109.138214 19448038 PMC2705016

[B20] Li H Durbin R Fast and accurate long-read alignment with Burrows-Wheeler transform Bioinformatics 2010 26 5 589 595 10.1093/bioinformatics/btp698 20080505 PMC2828108

[B21] Birol I Jackman SD Nielsen CB Qian JQ Varhol R Stazyk G Morin RD Zhao Y Hirst M Schein JE *De novo* transcriptome assembly with ABySS. Bioinformatics 2009 25 21 2872 2877 10.1093/bioinformatics/btp367 19528083

[B22] Childs KL Hamilton JP Zhu W Ly E Cheung F Wu H Rabinowicz PD Town CD Buell CR Chan AP The TIGR Plant Transcript Assemblies database Nucleic Acids Res 2007 35 Database issue D846 D851 17088284 10.1093/nar/gkl785 PMC1669722

[B23] Duvick J Fu A Muppirala U Sabharwal M Wilkerson MD Lawrence CJ Lushbough C Brendel V PlantGDB: a resource for comparative plant genomics Nucleic Acids Res 2008 36 Database issue D959 D965 18063570 10.1093/nar/gkm1041 PMC2238959

[B24] Cox MP Peterson DA Biggs PJ SolexaQA: At-a-glance quality assessment of Illumina second-generation sequencing data BMC Bioinformatics 2010 11 485 10.1186/1471-2105-11-485 20875133 PMC2956736

[B25] Punta M Coggill PC Eberhardt RY Mistry J Tate J Boursnell C Pang N Forslund K Ceric G Clements J The Pfam protein families database Nucleic Acids Res 2012 40 Database issue D290 301 22127870 10.1093/nar/gkr1065 PMC3245129

[B26] Birney E Clamp M Durbin R Genewise and genomewise Genome Res 2004 14 5 988 995 10.1101/gr.1865504 15123596 PMC479130

[B27] Li H Durbin R Fast and accurate short read alignment with Burrows-Wheeler transform Bioinformatics 2009 25 14 1754 1760 10.1093/bioinformatics/btp324 19451168 PMC2705234

[B28] Li H Handsaker B Wysoker A Fennell T Ruan J Homer N Marth G Abecasis G Durbin R 1000 Genome Project Data Processing Subgroup: the sequence alignment/map format and SAMtools. Bioinformatics 2009 25 16 2078 2079 10.1093/bioinformatics/btp352 19505943 PMC2723002

