# Supplementary figures and images for: Optimizing de novo common wheat transcriptome assembly using short-read RNA-Seq data
Source: BMC Genomics. 2012 Aug 14;13:392. doi: 10.1186/1471-2164-13-392 (PMC3485621; doi:10.1186/1471-2164-13-392)

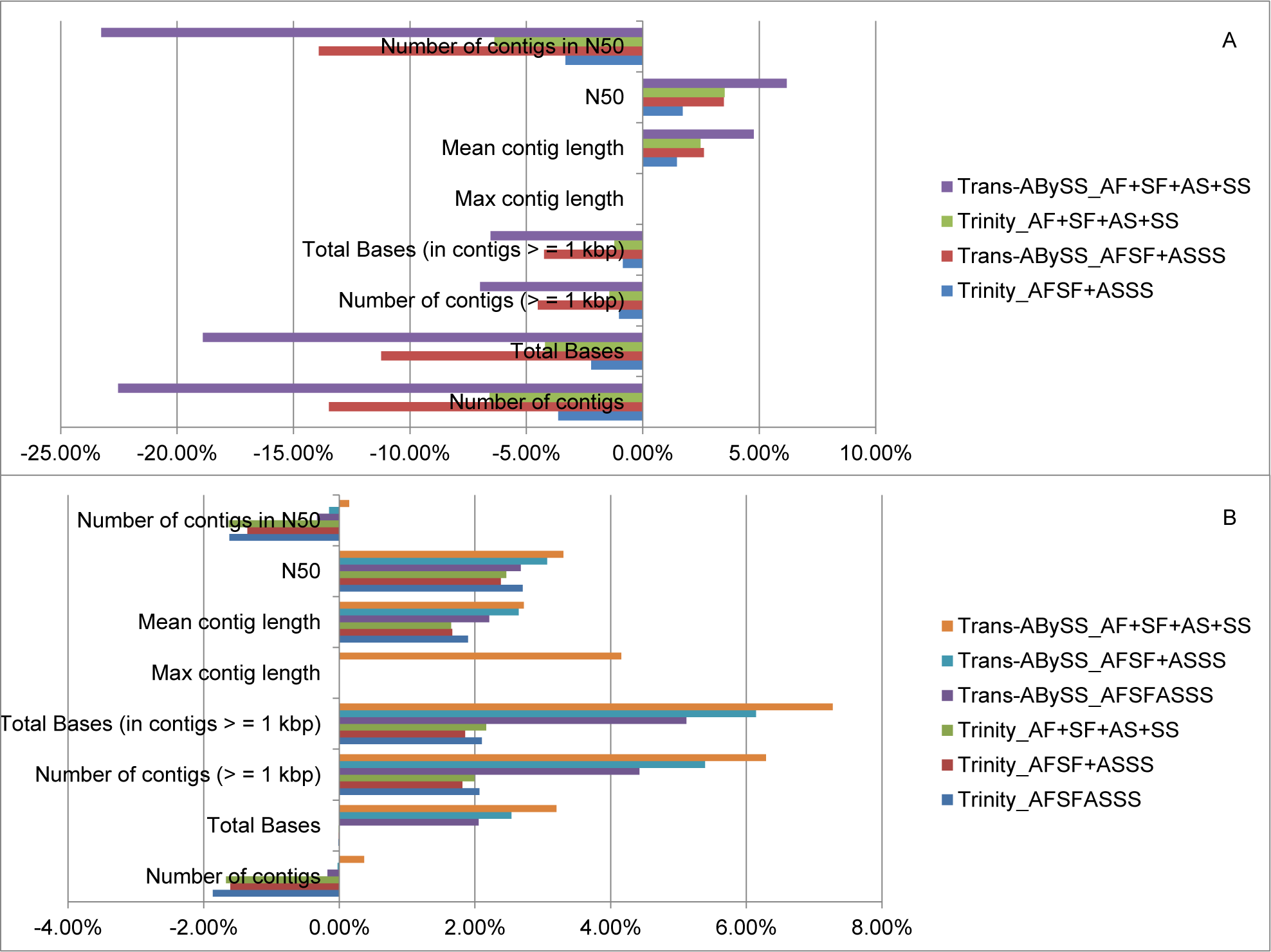

Supplement: Additional file 1 — Figure S1. Effects of merging assemblies of different samples and scaffolding (shown as changed proportions). [file 1471-2164-13-392-S1.tiff]
